# Clinical Impact of Atrial Fibrillation in Patients with Pulmonary Hypertension

**DOI:** 10.1371/journal.pone.0033902

**Published:** 2012-03-16

**Authors:** Dennis Rottlaender, Lukas J. Motloch, Daniela Schmidt, Sara Reda, Robert Larbig, Martin Wolny, Daniel Dumitrescu, Stephan Rosenkranz, Erland Erdmann, Uta C. Hoppe

**Affiliations:** 1 Department of Internal Medicine II, Paracelsus Medical University, Salzburg, Austria; 2 Department of Internal Medicine III, University of Cologne, Cologne, Germany; 3 Center of Molecular Medicine Cologne, University of Cologne, Cologne, Germany; University of Iowa Carver College of Medicine, United States of America

## Abstract

**Background:**

Pulmonary hypertension (PH) is associated with progressive impairment of right ventricular function, reduced exercise capacity and a poor prognosis. Little is known about the prevalence, clinical manifestation and impact of atrial fibrillation (AF) on cardiac function in PH.

**Methods:**

In a four year single-centre retrospective analysis 225 patients with confirmed PH of various origins were enrolled to investigate the prevalence of AF, and to assess the clinical manifestation, 6-minute walk distance, NT-proBNP levels, echocardiographic parameters and hemodynamics obtained by right heart catheterization in PH with AF.

**Results:**

AF was prevalent in 31.1%. In patients with PH and AF, parameters of clinical deterioration (NYHA/WHO functional class, 6-minute walk distance, NT-proBNP levels) and renal function were significantly compromised compared to patients with PH and sinus rhythm (SR). In the total PH cohort and in PH not related to left heart disease occurrence of AF was associated with an increase of right atrial pressure (RAP) and right atrial dilatation. While no direct association was found between pulmonary artery pressure (PAP) and AF in these patients, right ventricular function was reduced in AF, indicating more advanced disease. In PH due to left heart failure the prevalence of AF was particularly high (57.7% vs. 23.1% in other forms of PH). In this subgroup, left atrial dilatation, increase of pulmonary capillary wedge pressure, PAP and RAP were more pronounced in AF than in SR, suggesting that more marked backward failure led to AF in this setting.

**Conclusion:**

PH is associated with increased prevalence of AF. Occurrence of AF in PH indicates clinical deterioration and more advanced disease.

## Introduction

Pulmonary hypertension (PH) – i.e. an elevated mean pulmonary artery pressure (PAP), ≥25 mmHg at rest – defines a group of diseases characterized by a progressive increase in pulmonary vascular resistance leading to right ventricular failure and premature death [1], [2], [3]. Based on the pathophysiological mechanisms and etiology, the current clinical classification distinguishes five groups of PH [4]. Left heart failure (HF) is one common cause of PH, representing group 2 of the Dana-Point classification. Left ventricular systolic dysfunction, diastolic dysfunction or valvular disease may all result in elevated PAP. In fact, PH is being found in more than 60% of patients with moderate or severe HF [5]. However, from a pathophysiological point of view and with regard to therapeutic options, PH due to left heart disease is clearly differentiated from pulmonary arterial hypertension (PAH) and has to be appreciated as a separate entity. At present, targeted PAH therapies are not recommended for this subgroup.

Atrial fibrillation (AF) is the most common chronic arrhythmia. Chronic left heart failure and AF often coexist. Both are responsible for increased mortality, more frequent hospitalizations, reduced exercise capacity, decreased quality of life and substantial health care expenditures [6]. In addition to merely having risk factors in common, AF and heart failure are believed to directly predispose to each other [7], [8]. The risk of developing AF during long-term follow-up appears to be 5 to 10 times higher in patients with left heart failure than in healthy persons [9], [10], [11], [12]. Some studies have shown that the onset of AF in these patients is associated with clinical and hemodynamic deterioration due to loss of atrial contractility, tachycardia, and lack of atrioventricular synchrony, as well as a worse long-term prognosis [13], [14].

Although the association between AF and left heart failure is well documented, the predisposing factors for developing AF in this setting are not fully understood. Moreover, the prevalence of AF in PH with or without compromised right ventricular function has not been defined. Learning more about which types of patients with PH develop AF may yield important insights into the pathogenesis of AF in this condition, and importantly may help guide clinicians in the monitoring, evaluation, and management of these patients.

## Methods

### Study participants

The study was performed according to good clinical practice and in compliance with the Helsinki declaration. Individual patient were not identified. An individual written consent was obtained by every patient, which is usually performed due to quality management issues in our hospital. The study and study design was approved by the institutional review board. The study cohort comprised 225 consecutive patients with confirmed diagnosis of PH referred to a single-centre between October 01, 2006 and March 31, 2010. In all eligible patients, exact classification of PH into one of the five groups according to the Dana-Point classification was performed [4], and information about the clinical severity (NYHA/WHO functional class), medication, concomitant diseases, 6-minute walk distance and N-terminal pro-brain natriuretic peptide (NT-proBNP) levels were obtained from the University Patient Database. Furthermore, if available, echocardiography was analyzed. Patients were divided into two groups: 1. patients with PH and sinus rhythm (PH-SR) and 2. patients with PH and atrial fibrillation (PH-AF). Given distinct cardiac pathomechanisms, subgroup analysis was performed in patients with PH due to left heart failure (a. PH-HF SR and b. PH-HF AF) and PH due to any other cause (c. PH-nonHF SR and d. PH-nonHF AF).

### Definition of prevalent AF

“Prevalent AF” was defined as the presence of AF on electrocardiogram during the index hospitalization and/or as indicated by a diagnosis found in medical records, the hospitalization database, or ambulatory visit databases. “Electrocardiographic AF” was defined as the presence of an irregular rhythm with fibrillatory waves and no defined P-waves [9]. Diagnoses were based on physician-assigned diagnoses in the medical records and/or the presence of corresponding ICD-9-CM codes for AF (427.31) in the hospital discharge or ambulatory visit clinical databases. Atrial fibrillation was sub-classified into paroxysmal AF or chronic AF (persistent or permanent) according to international guidelines.

### Etiology and severity of PH

Patients were classified according to the Dana Point classification of PH, and clinical severity was assessed according to the WHO functional classes for PH [4]. In PH due to left heart failure with reduced ejection fraction, left ventricular ejection fraction was defined as lower than 40% (as assessed by echocardiography, biplane Simpson method in apical four chamber view). Heart failure with preserved ejection fraction was diagnosed following the consensus statement of the Association of the European Society of Cardiology [15]. Valvular disease was defined as mitral and/or aortic stenosis or insufficiency or valve repair.

### Six-minute walk testing

Patients were instructed to walk down a 100-foot corridor at their own pace, attempting to cover as much ground as possible. At the end of the six minute interval the total distance was determined. The test was performed by personnel that had been trained according to the current ATS consensus statement on six-minute walk testing [16]. Forty-two patients were excluded from testing due to disability of movement.

### Clinical laboratory parameters

N-terminal pro-brain natriuretic peptide (NT-proBNP) was measured in every patient as a marker of heart failure known to correlate with survival and the severity of disease in both left and right heart failure. Additionally, renal function parameters, i.e. creatinine, urea nitrogen and estimated glomerular filtration rate (eGFR, using the MDRD equation [17]) were determined.

### Echocardiography

All transthoracic echocardiographic studies were obtained by experienced investigators using a Philips iE 33 echocardiography system (Philips, Hamburg, Germany). Left atrial diameter (edge-to-edge method, parasternal view), right atrial area (measured at end-systole in the apical four chamber view), left ventricular ejection fraction (biplane Simpson method), Tricuspid Annular Plane Systolic Excursion (TAPSE) and pulmonary artery systolic pressure were recorded according to current recommendations [18]. Only complete datasets were included in the statistical analysis.

### Right heart catheterization

Right heart catheterization was performed via the femoral vein using a balloon flotation catheter (PWP catheter, Medtronic, Minneapolis, USA). Fluoroscopic guidance was used to cannulate the pulmonary artery and obtain pulmonary capillary wedge position. Right heart catheterization studies were analyzed for systolic and mean pulmonary arterial pressure, pulmonary capillary wedge pressure (PCWP), right atrial pressure and pulmonary vascular resistance. Cardiac output was estimated using the Fick technique. Echocardiograms and right heart catheterization were performed at the same time.

### Statistical analysis

Statistical analyses were performed using PASW statistics 18 software (SPSS, Chicago, USA). All variables were tested for normal distribution with the Kolmogorov-Smirnov test. The results are given as mean ± standard error of mean (SEM). All groups and subgroups were compared for PH classification, clinical manifestation, 6-minute-walk-testing, laboratory parameters, data of echocardiography and right heart catheterization. Differences between groups and subgroups were evaluated by chi-square-testing for discrete variables and student-t test for continuous variables. For ordinal data Mann-Whitney-U test was used. A p<0.05 was considered as statistically significant.

## Results

### Baseline characteristics and prevalence of AF

A total of 225 patients with PH were analyzed in this retrospective study. Seventy patients (31.1%) of the total study cohort had evidence of AF. In patients with AF, 41.3% had paroxysmal AF, whereas 58.7% presented with chronic AF. The demographic variables of the individual groups with and without AF are shown in table 1. Patients in both groups were predominantly female. Mean age did not differ significantly between the PH-SR and PH-AF group.

**Table 1:** Patients' Characteristics.

	PH-SR(n = 155)		PH-AF(n = 70)	
Characteristic	n	% or Mean ± SEM	n	% or Mean ± SEM
Age	155	62.9±1.2	70	71.2±1.1
Male	58	37.4%	25	35.7%
Female	97	62.6%	45	64.3%
Mean heart rate (bpm)	155	79.5±1.3	70	74.6±1.9
**WHO group | PH subgroup**				
Pulmonary arterial hypertension	78	50.3%	32	45.7%
Idiopathic	31	20.0%	24	34.3%*
Heritable	2	1.3%	0	0%
Drug- and toxin-induced	4	2.6%	0	0%
Associated with				
Congenital heart disease	6	3.9%	3	4.3%
HIV infection	3	1.9%	0	0%
Connective tissue disease	32	20.6%	3	4.3%*
Portal hypertension	0	0%	2	2.9%
Veno-occlusive disease	0	0%	0	0%
Pulmonary hypertension due to left heart failure	22	14.2%	30	42.9%*
Systolic dysfunction	6	3.9%	10	14.3%*
Diastolic dysfunction	13	8.4%	17	24.3%*
Valvular disease	3	1.9%	3	4.3%
Pulmonary hypertension due to pulmonary disease	21	13.5%	5	7.1%
Chronic obstructive pulmonary disease	7	4.5%	3	4.3%
Interstitial lung disease	14	9.0%	2	2.9%
Chronic thromboembolic pulmonary hypertension	31	20.0%	2	2.9%*
Others	3	1.9%	1	1.4%
**Medication**				
Phosphodiesterase-5-inhibitor	51	32.9%	31	44.3%
Endothelin-1 antagonist	47	30.3%	15	21.4%
Prostacyclin	7	4.5%	3	4.3%
Calcium channel blockers	29	18.7%	17	24.3%
Betablocker	65	41.9%	51	72.9%*
Digitalis	8	5.2%	26	37.1%*
Amiodarone	1	0.6%	7	10.0%*
Sotalol	2	1.3%	1	1.4%
Diuretics	112	72.3%	65	92.9%*
Angiotensin receptor blockers/AT-1 antagonist	82	52.9%	42	60.0%
Cumarine	61	39.4%	55	78.6%*
Acetylsalicylic acid	49	31.6%	11	15.7%*
Clopidogrel	10	6.5%	4	5.7%
Statins	41	26.5%	27	38.6%
Nitrates	11	7.1%	3	4.3%
**Concomitant disease**				
Coronary artery disease	39	25.2%	19	27.1%
Myocardial infarction	9	5.8%	8	11.4%
Coronary artery bypass graft	5	3.2%	7	10.0%
Dilated cardiomyopathy	3	1.9%	7	10.0%*
Valvular disease	14	9.0%	20	28.6%*
Arterial hypertension	75	48.4%	45	64.3%*
Pulmonary disease	97	62.6%	27	38.6%*

* p<0.05 vs. PH-SR.

When evaluating the relative percentage of the distinct etiologies of PH according to the Dana Point classification in the PH-AF group versus PH-SR group, we obtained no significant difference for PH due to pulmonary disease. However, PH due to left heart failure (PH-HF) was markedly more common in the PH-AF group (PH-AF 42.9% vs. PH-SR 14.2%, p<0.05). This observation was consistent for all causes of left heart failure, though for valvular disease the difference did not reach statistical significance most likely due to the limited number of patients in this subgroup (table 1). Notably, 57.7% of all patients with PH-HF presented with AF, compared to 23.1% in the PH-nonHF group. Conversely, the relative percentage of chronic thromboembolic pulmonary hypertension (CTEPH) was higher in the PH population with SR (PH-SR 20.0% vs. PH-AF 2.9%, p<0.05). While we observed a similar percentage of pulmonary arterial hypertension (PAH) per se in the PH-AF and PH-SR group (45.7% vs. 50.3%, n.s.), idiopathic PAH was more frequent in those with AF (PH-AF 34.3% vs. PH-SR 20.0%, p<0.05), reflecting an AF prevalence of 43.6% in this subpopulation.

A comparison of patients with paroxysmal (PH-AF paroxysmal) and chronic (PH-AF chronic) AF in PH revealed chronic AF to be associate with PH due to pulmonary disease (PH-AF paroxysmal 0% vs. PH-AF chronic 11.9%, p<0.05). Moreover, PH due to systolic dysfunction was associated with chronic AF, while diastolic dysfunction was related to paroxysmal AF (systolic dysfunction: PH-AF paroxysmal 3.6% vs. PH-AF chronic 21.4%, p<0.05; diastolic dysfunction: PH-AF paroxysmal 32.1% vs. PH-AF chronic 19.0%, p<0.05).

Targeted therapy for PAH such as prostacyclin analogues, endothelin receptor antagonists and phosphodiesterase-5 inhibitors were equally prescribed in PAH patients with and without AF. Expectedly, treatment for AF, i.e. betablockers, digitalis, amiodarone and cumarine was more common in the PH-AF group. Notably, patients with PH-AF more often received diuretics indicating more advanced heart failure.

### Clinical presentation of patients with AF in PH

**Figure 1:**
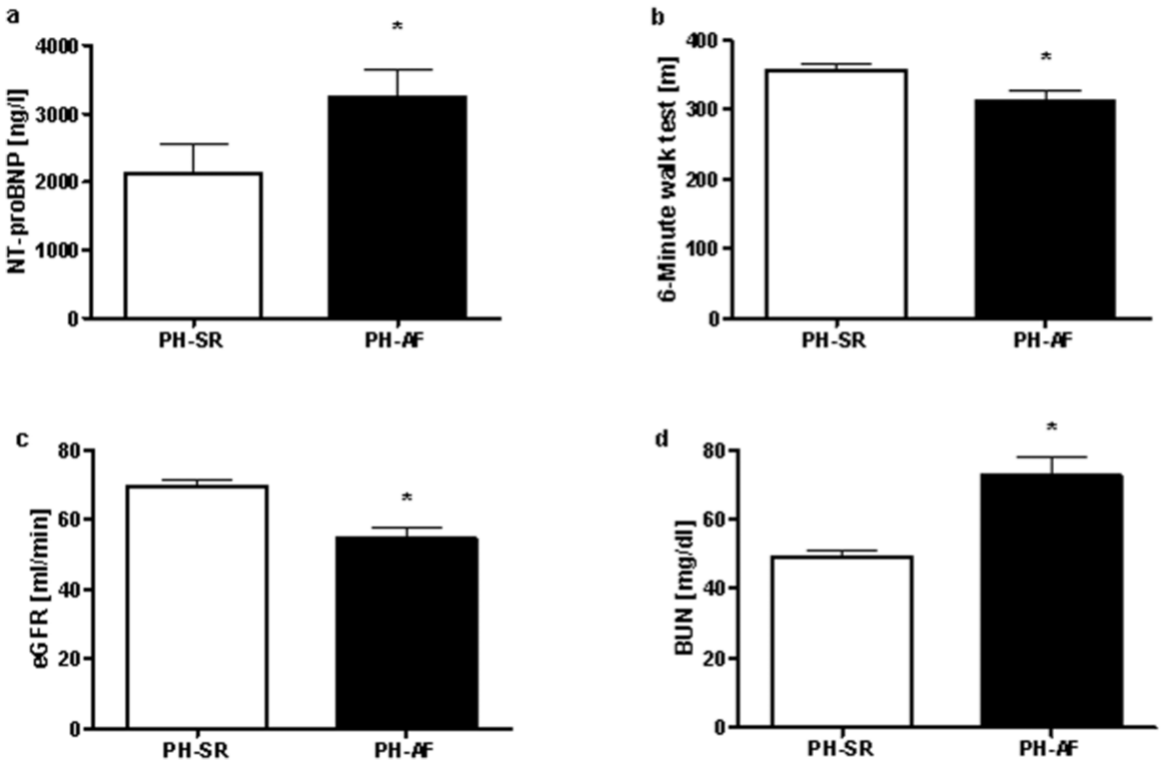
Laboratory parameters and exercise capacity in PH with and without AF. NT-pro-BNP, estimated glomerular filtration rate (eGFR), urea nitrogen (BUN) and 6-minute walk distance of patients with PH were compared in those with AF (PH-AF) and SR (PH-SR). * p<0.05. Error bars representing standard error of mean.

Thus far, clinical manifestation of AF in PH has not been analyzed systematically. Therefore, we evaluated functional class, exercise capacity, and laboratory parameters indicative of hemodynamic status in this population. The clinical condition in patients with AF in PH was more severe than in patients without AF, as indicated by the NYHA/WHO functional class (table 2). Consistently, the 6-minute walk distance was significantly shorter in the PH-AF group (PH-SR vs. PH-AF: 355.55±9.86 m, n = 130 vs. 321.98±14.1 m, n = 53; p<0.05, figure 1). Moreover, in patients with AF, the elevation of NT-proBNP serum levels was more pronounced (PH-SR vs. PH-AF: 2128.88±429.97 ng/l, n = 155 vs. 3252.79±401.76 ng/l, n = 70; p<0.05, figure 1).

**Table 2:** Effect of AF on NYHA class and renal function in PH.

	PH-SR		PH-AF	
NYHA classification	n	%	n	%
NYHA I	2	1.3%	0	0%#
NYHA II	52	33.5%	17	24.3%#
NYHA III	98	63.2%	51	72.9%#
NYHA IV	3	1.9%	2	2.9%#
**Renal function**				
CKD class I	31	20.0%	3	4.3%#
CKD class II	62	40.0%	23	32.9%#
CKD class III	55	35.5%	35	50.0%#
CKD class IV	6	3.9%	8	11.4%#
CKD class V	1	0.6%	1	1.4%#

CKD = Chronic kidney disease classification.

# p<0.05 vs. PH-SR.

Given that renal failure was shown to correlate with reduced survival and clinical deterioration, standard parameters of renal function (creatinine, urea nitrogen, eGFR) and chronic renal failure classification were analyzed. As shown in figure 1, AF was associated with impaired renal function, reflected by a significant increase of creatinine and urea nitrogen in patients suffering from AF compared to those without AF (PH-SR vs. PH-AF: 1.07±0.03 mg/dl, n = 154 vs. 1.37±0.11 mg/dl, n = 73; 49.17±1.97 mg/dl, n = 154 vs. 72.61±5.44 mg/dl, n = 69; p<0.05), and a reduced eGFR (PH-SR vs. PH-AF: 69.68±2.11 ml/min, n = 154 vs. 54.96±2.65 ml/min, n = 69; p<0.05). Accordingly, patients with AF in PH were found to be in more severe stages of chronic renal failure (table 2).

### Hemodynamic parameters in PH with AF

**Figure 2:**
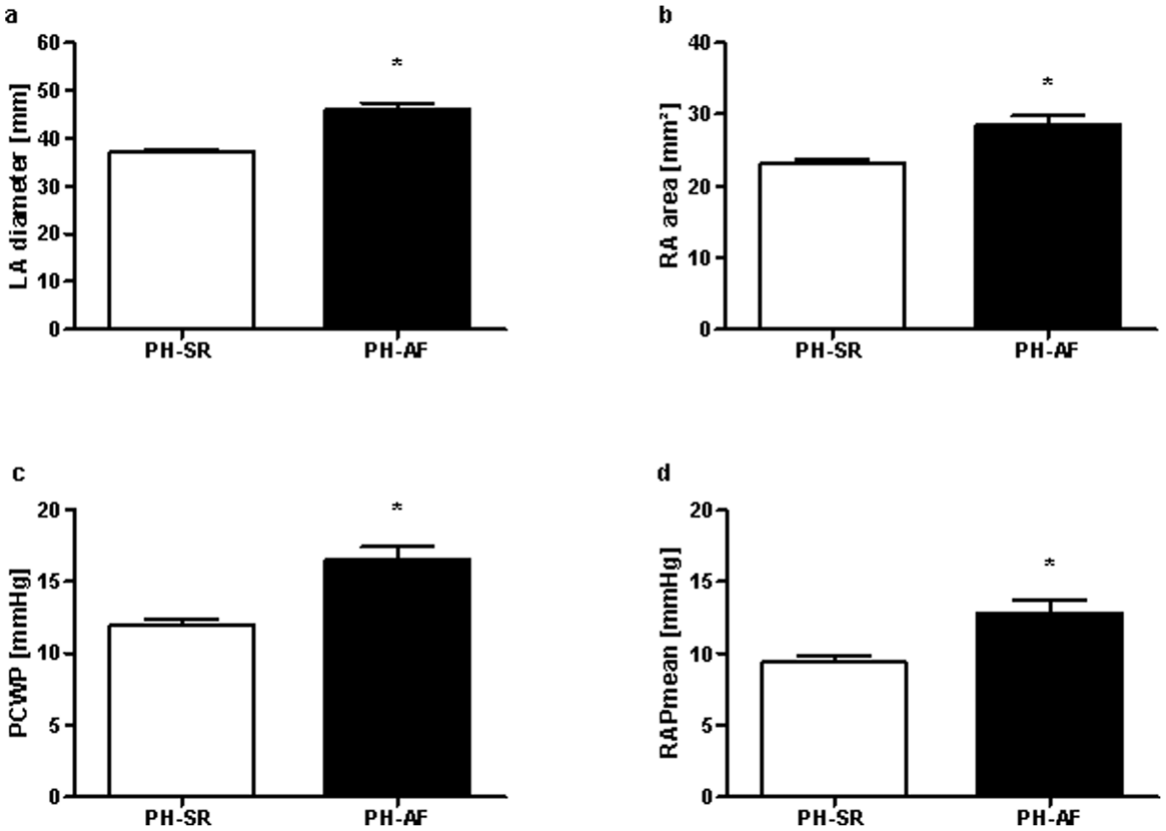
Hemodynamic parameters associated with AF in PH. Left atrial (LA) diameter and right atrial (RA) area were measured by echocardiography in PH-AF compared to PH-SR. PCWP and mean right atrial pressure (RAPmean) were obtained by right heart catheterization in the presence (PH-AF) or absence (PH-SR) of AF in patients with PH. * p<0.05.

Cardiac function and hemodynamic data were evaluated by echocardiography and right heart catheterization (table 3). Expectedly, left atrial diameter was significantly larger, pulmonary capillary wedge pressure (PCWP) was higher (figure 2, table 3), and left ventricular ejection fraction was reduced in the PH-AF group, reflecting the marked fraction of PH due to left heart disease.

**Table 3:** Effect of AF on hemodynamic parameters in PH.

	PH-SR		PH-AF	
Echocardiography	n	Mean ± SEM	n	Mean ± SEM
Left atrial diameter [mm]	154	37.09±0.64	69	46.16±1.28*
Right atrial area [mm^2^]	154	23.15±0.62	69	28.59±1.19*
TAPSE [mm]	154	20.97±0.48	69	18.07±0.68*
Systolic pulmonary artery pressure [mmHg]	154	64.59±1.82	69	60.10±2.18
Left ventricular ejection fraction [%]	154	63.81±0.55	69	59.84±1.52*
**Right heart catheterization**				
Systolic pulmonary artery pressure [mmHg]	155	65.00±1.86	70	67.51±2.34
Mean pulmonary artery pressure [mmHg]	155	40.25±1.12	70	40.80±1.56
Mean right atrial pressure [mmHg]	135	9.4±0.43	61	12.85±0.86*
PCWP [mmHg]	155	11.96±0.38	70	16.50±0.90*
Pulmonary vascular resistance [Wood units]	146	7.29±0.42	66	6.45±0.54
Cardiac output [l/min]	127	4.36±0.12	47	3.91±0.16*

* p<0.05 vs. PH-SR.

Notably, systolic and mean pulmonary artery pressures showed no differences in the PH-AF compared to the PH-SR group, implicating that pulmonary artery pressure per se has no direct effect on the occurrence of AF or vice versa. However, we obtained a significant increase of the right atrial area and mean right atrial pressure in PH with versus without AF (figure 2, table 3). Moreover, right ventricular function assessed by TAPSE was significantly reduced in PH-AF. Consistently, cardiac output was lower in the PH-AF group compared to the PH-SR group (table 3).

A comparison of patients with paroxysmal (PH-AF paroxysmal) and chronic (PH-AF chronic) AF in PH indicated increased right atrial area and mean right atrial pressure in patients with chronic AF versus paroxysmal AF (PH-AF paroxysmal 25.04±1.46 mm^2^ and 10.69±0.98 mmHg vs. PH-AF chronic 30.93±1.36 mm^2^ and 14.91±1.03 mmHg, p<0.05).

### AF in PH not related to left heart disease

Given the distinct pathopyhsiology of pre- versus postcapillary PH, in a subanalysis patients with PH due to left heart disease (PH-HF, group 2) were separated from patients with PH due to any other cause (PH-nonHF, groups 1, 3, 4, or 5). In PH not related to left heart disease, AF was observed in 23.1% (table 4). In PAH (group 1) AF was found in 29.1% (table 5). AF was associated with clinical deterioration, as indicated by a higher NYHA/WHO functional class, shorter 6-minute walk distance, more severely elevated NT-proBNP serum levels and compromised renal function compared to patients in SR (table 4, table 5).

**Table 4:** Comparison of subgroups with SR vs. AF in PH not related to left heart disease (nonHF).

	PH-SR nonHF		PH-AF nonHF	
Echocardiography	n	% or Mean ± SEM	n	% or Mean ± SEM
Left atrial diameter [mm]	132	37.05±0.68	39	43.31±1.55*
Right atrial area [mm^2^]	132	23.53±0.66	38	30.51±1.61*
TAPSE [mm]	132	20.74±0.52	38	18.35±0.87*
Systolic pulmonary artery pressure [mmHg]	132	66.67±1.98	39	65.72±3.15
Left ventricular ejection fraction [%]	132	64.52±0.43	40	64.30±1.16
**Right heart catheterization**				
Systolic pulmonary artery pressure [mmHg]	133	67.02±2.04	40	68.25±3.24
Mean pulmonary artery pressure [mmHg]	133	41.25±1.23	40	40.38±2.04
Mean right atrial pressure [mmHg]	115	8.93±0.45	36	11.03±0.63*
PCWP [mmHg]	133	10.86±0.30	40	12.35±0.63
Pulmonary vascular resistance [Wood units]	126	7.74±0.46	38	7.16±0.75
Cardiac output [l/min]	111	4.36±0.13	25	3.94±0.19
**Laboratory parameters**				
NT-proBNP [pg/l]	133	1738.86±212.05	40	3449.40±547.61*
Creatinine [mg/dl]	133	1.06±0.03	40	1.23±0.08*
Urea nitrogen [mg/dl]	133	48.23±2.10	40	64.40±5.39*
eGFR [ml/min//1.72 m^2^]	133	70.78±2.34	40	58.18±3.51*
**NYHA classification**				
NYHA I	1	0.8%	0	0%#
NYHA II	51	38.3%	8	20.0%#
NYHA III	78	58.6%	31	77.5%#
NYHA IV	3	2.3%	1	2.5%#
**6-minute walk test**				
6-minute walk test [m]	113	364.67±10.51	31	303.87±17.36*
**Renal function**				
CKD class I	28	21.1%	2	5.0%#
CKD class II	56	42.1%	15	37.5%#
CKD class III	43	32.3%	20	50.0%#
CKD class IV	6	4.5%	3	7.5%#
CKD class V	0	0%	0	0%#

CKD = Chronic kidney disease classification, eGFR = estimated glomerular filtration rate.

* p<0.05 vs. PH-SR (student t-test).

# p<0.05 vs. PH-SR (Mann-Whitney test).

**Table 5:** Comparison of subgroups with SR vs. AF in PAH.

	PAH-SR		PAH-AF	
Echocardiography	n	% or Mean ± SEM	n	% or Mean ± SEM
Left atrial diameter [mm]	77	37.04±0.81	31	43.61±1.53*
Right atrial area [mm^2^]	77	22.92±0.87	31	31.33±1.49*
TAPSE [mm]	77	20.99±0.65	31	17.74±0.94*
Systolic pulmonary artery pressure [mmHg]	77	62.83±2.62	31	66.58±3.38
Left ventricular ejection fraction [%]	77	64.49±0.49	32	64.06±1.40
**Right heart catheterization**				
Systolic pulmonary artery pressure [mmHg]	78	65.27±2.31	32	70.03±3.83
Mean pulmonary artery pressure [mmHg]	78	41.22±1.73	32	41.47±2.45
Mean right atrial pressure [mmHg]	66	8.90±0.49	32	11.43±0.65*
PCWP [mmHg]	78	10.53±0.38	32	12.84±0.63
Pulmonary vascular resistance [Wood units]	74	7.49±0.60	32	7.58±0.87
Cardiac output [l/min]	66	4.41±0.19	21	3.98±0.26
**Laboratory parameters**				
NT-proBNP [pg/l]	78	1705.81±232.49	32	3529.84±537.77*
Creatinine [mg/dl]	78	1.06±0.04	32	1.38±0.09*
Urea nitrogen [mg/dl]	78	45.98±2.38	32	68.19±4.55*
eGFR [ml/min//1.72 m^2^]	78	73.76±3.47	32	59.09±3.09*
**NYHA classification**				
NYHA I	1	1.3%	0	0%#
NYHA II	35	44.9%	7	21.9%#
NYHA III	41	52.6%	24	75.0%#
NYHA IV	1	1.3%	1	3.1%#
**6-minute walk test**				
6-minute walk test [m]	66	384.21±11.67	26	311.35±18.04*
**Renal function**				
CKD class I	20	25.6%	2	6.3%#
CKD class II	30	38.5%	13	40.6%#
CKD class III	24	30.8%	15	46.9%#
CKD class IV	4	5.1%	2	6.3%#
CKD class V	0	0%	0	0%#

CKD = Chronic kidney disease classification, eGFR = estimated glomerular filtration rate.

* p<0.05 vs. PAH-SR (student t-test).

# p<0.05 vs. PAH-SR (Mann-Whitney test).

While consistent with the total PH cohort, no difference of systolic and mean pulmonary arterial pressures in the presence or absence of AF was obtained; increased right atrial pressure and size as well as reduced right ventricular function were the most obvious hemodynamic differences between prevalent AF versus no AF in PH-nonHF and PAH. As expected, left ventricular ejection fraction and PCWP were similar in both groups (table 4, table 5).

### AF in left heart disease with PH

In patients with PH due to left heart disease, AF was prevalent in 57.7%. While AF in this cohort did not further diminish exercise capacity or NYHA/WHO functional class, we still observed higher NT-proBNP values and more severely compromised renal function in those patients with AF versus SR (table 6).

**Table 6:** Comparison of subgroups with SR vs. AF in PH related to left heart disease (HF).

	PH-SR HF		PH-AF HF	
Echocardiography	n	% or Mean ± SEM	n	% or Mean ± SEM
Left atrial diameter [mm]	22	37.36±1.89	30	49.87±1.96*
Right atrial area [mm^2^]	22	20.87±1.65	30	26.17±1.69*
TAPSE [mm]	22	22.32±1.25	29	17.45±0.94*
Systolic pulmonary artery pressure [mmHg]	22	52.14±3.75	30	52.80±2.34
Left ventricular ejection fraction [%]	22	59.55±2.69	29	53.69±2.90
**Right heart catheterization**				
Systolic pulmonary artery pressure [mmHg]	22	52.86±3.29	30	64.10±3.36*
Mean pulmonary artery pressure [mmHg]	22	34.23±2.01	30	41.37±2.46*
Mean right atrial pressure [mmHg]	20	12.10±1.16	25	16.92±1.37*
PCWP [mmHg]	22	18.63±1.21	30	22.13±1.37
Pulmonary vascular resistance [Wood units]	20	4.48±0.77	28	5.50±0.77
Cardiac output [l/min]	16	4.33±0.26	22	3.89±0.28
**Laboratory parameters**				
NT-proBNP [pg/l]	22	1149.00±181.61	30	3257.30±568.65*
Creatinine [mg/dl]	21	1.11±0.06	29	1.58±0.25*
Urea nitrogen [mg/dl]	21	55.10±5.60	29	83.93±10.35*
eGFR [ml/min//1.72 m^2^]	21	62.71±4.27	29	50.52±3.97*
**NYHA classification**				
NYHA I	2	9.1%	0	0.0%
NYHA II	4	18.2%	11	36.7%
NYHA III	16	72.7%	19	63.3%
NYHA IV	0	0%	0	0%
**6-minute walk test**				
6-minute walk test [m]	17	294.88±24.29	22	333.41±23.99
**Renal function**				
CKD class I	3	13.6%	1	3.3%#
CKD class II	8	36.4%	8	26.7%#
CKD class III	10	45.5%	15	50.0%#
CKD class IV	0	0%	5	16.7%#
CKD class V	1	4.5%	1	3.3%#

CKD = Chronic kidney disease classification, eGFR = estimated glomerular filtration rate.

* p<0.05 vs. PH-SR (student t-test).

# p<0.05 vs. PH-SR (Mann-Whitney test).

Left atrial size was significantly larger and PCWP tended to be higher in prevalent AF, although the latter did not reach statistical significance. Invasive measurements demonstrated increased systolic and mean pulmonary arterial and right atrial pressures associated with AF. Moreover, echocardiography indicated right heart impairment, i.e. increased right atrial area and suppressed TAPSE in the subgroup with prevalent AF (table 6).

## Discussion

Atrial fibrillation affects 1–2% of the general population. The prevalence of AF increases with age, from 0.5% at 40–50 years, to 5–15% at 80 years [19]. Atrial fibrillation is highly prevalent among patients with left ventricular heart failure, and can lead to adverse consequences, including tachycardia-related cardiomyopathy, reduction in left ventricular preload attributable to disorganized atrial contractions, increased risk of systemic embolism, and overall poorer long-term outcome [20], [21], [22]. Supraventricular tachyarrhythmias occurred in patients with pulmonary hypertension with an annual incidence of 2.8%. Atrial flutter and atrial fibrillation were equally common and both arrhythmias led to acute clinical deterioration with signs of right heart failure, while only atrial fibrillation exerted an impact on mortality [23]. However, little is known about the total prevalence of AF in patients with PH and possible differences among distinct etiological groups of PH have not been defined. In the present cohort of patients with confirmed PH of various origins, we found that AF affected approximately one-third of patients. Thus, the prevalence of AF in PH is considerably higher than in the normal population at similar age [24]. This was also true for all PH subgroups except for patients with chronic thromboembolic pulmonary hypertension (CTEPH).

Particularly, more than half of the patients with PH related to left heart disease were affected by AF. Symptomatic heart failure has been reported in 30% of AF patients and AF is found in up to 30% of heart failure patients, depending on the underlying cause and severity of heart failure [9], [19]. Pulmonary hypertension in heart failure is associated with a poor prognosis and an increased severity of disease [5]. Compared to the cited populations mentioned above we observed a significantly higher prevalence of AF in patients with left heart disease combined with PH (57.7%), supporting the notion that our patients suffered from more severe heart failure, which was also indicated by the high prevalence of patients with NYHA III and markedly elevated NT-proBNP levels. Moreover, these data suggest that more advanced heart failure leading to PH is a relevant risk factor for the development of AF. Given the retrospective design of our study, we may even have underestimated the true prevalence of AF in PH. Diagnosis of AF was made by carefully analyzing patient's history and standard electrocardiograms. Because of the lack of other means of rhythm monitoring (i.e. periodical Holter recordings, implanted event recorders), it is likely that some self-limiting silent AF episodes might have been missed.

When analyzing hemodynamic factors promoting AF in PH it seems plausible to separately evaluate patients with and without left heart disease. Previously, reduced left ventricular function, elevated end-diastolic left ventricular pressure and, thus, higher PCWP and larger left atrial diameter have been associated with an increased propensity of AF in left heart disease. Consistently, in the present study these parameters were more severely altered in prevalent AF than in patients with SR. Notably, in PH-HF with AF we observed increased pulmonary artery pressures in invasive measurements, which are more accurate than echocardiographic estimation [25], and signs of right heart impairment, indicating that AF in PH related to left heart disease was associated with more marked backward failure compared to SR.

Thus far, hemodynamic factors that might contribute to onset of AF in PH not related to left heart disease have not been evaluated. In the present analysis, elevated right atrial pressure and right atrial dilatation were the most prominent parameters associated with prevalent AF in PH-nonHF. While systolic and mean pulmonary pressures did not directly correlate with AF occurrence in nonHF patients, the severity of pulmonary hypertension might have been masked by impairment of right ventricular function, thus rather supporting the notion that AF is more common in more advanced PH. These results provide insight into the possible pathophysiology of AF in PH and indicate a different pathomechanism of AF induction in PH with versus without left heart disease. In the absence of left heart disease (i) left atrial pressure does not play a pathophysiological role and (ii) pulmonary artery pressure does not seem to provoke AF by itself, but an increase in right atrial pressure leading to right atrial dilatation seems to be responsible for onset of AF.

There is only limited data available regarding the clinical consequences of AF in patients with various forms of PH. Previous studies indicated that elevated heart rate might lead to increased mortality in patients suffering from PH. Notably, mean heart frequency in PH-AF was not significantly different compared to PH-SR in our population.

The influence of AF on clinical performance and cardiac function in PH has not been investigated yet. In our cohort patients with PH-AF demonstrated significant impairment in NYHA/WHO functional class and 6-minute walk distance compared to PH-SR. This clinical deterioration in the presence of AF was also evident by the higher prescription rate of diuretics. Elevation of NT-proBNP as a marker for heart failure and renal insufficiency have been implicated to correlate with prognosis and severity of disease in PH. In the present study these laboratory parameters were consistently increased in the total PH population and in all subgroups with prevalent AF compared to SR, further supporting the notion that AF in PH is associated with more advanced compromise of hemodynamic function.

Recently, renal failure, reduced 6-minute walk distance, elevated mean right atrial pressure, and increased brain natriuretic peptide have been suggested as independent predictors of mortality in PH. Therefore, it remains to be determined in a larger prospective study whether AF in PH is just a marker of more advanced disease, is an independent risk factor, and/or if cardioversion might improve symptoms, exercise capacity and possibly prognosis in this population.
